# Attractiveness Evaluation and Identity of Self-face: The Effect of Sexual Dimorphism

**DOI:** 10.1177/20416695211058799

**Published:** 2021-12-03

**Authors:** Zhaoyi Li, Xiaofang Lei, Xinze Yan, Zhiguo Hu, Hongyan Liu

**Affiliations:** 1 Department of Psychology, 12646 Zhejiang Sci-Tech University, Hangzhou, P. R. China; 2 Center for Cognition and Brain Disorders, 26494 The Affiliated Hospital of Hangzhou Normal University, Hangzhou, P. R. China; 3 Zhejiang Key Laboratory for Research in Assessment of Cognitive Impairments, Hangzhou, P. R. China

**Keywords:** self-face, sexual dimorphism, attractiveness, similarity

## Abstract

The present study aims to explore the influence of masculine/feminine changes on the attractiveness evaluation of one's own face, and examine the relationship of this attractiveness evaluation and the similarities between masculine/feminine faces and original faces. A picture was taken from each participant and considered as his or her original self-face, and a male or female face with an average attractiveness score was adopted as the original other face. Masculinized and feminized transformations of the original faces (self-face, male other face, and female other face) into 100% masculine and feminine faces were produced with morphing software stepping by 2%. Thirty female participants and 30 male participants were asked to complete three tasks, i.e., to “like” or “not like” the original face judgment of a given face compared to the original face, to choose the most attractive face from a morphed facial clip, and to subjectively evaluate the attractiveness and similarity of morphed faces. The results revealed that the acceptable range of masculine/feminine transformation for self-faces was narrower than that for other faces. Furthermore, the attractiveness ratings for masculinized or femininized self-faces were correlated with the similarity scores of the faces with the original self-faces. These findings suggested that attractiveness enhancement of self-face through masculinity/femininity must be within reasonable extent and take into account the similarity between the modified faces and the original self-face.

## Introduction

Every time we look in the mirror, we clearly know that we are looking at ourselves. Our own face is special and unique to us. It is the crucial carrier of our self-concept and emotional state (Catherine & Raffard, 2018; Sforza et al., 2010; Sugiura et al., 2012; Yoon & Kircher, 2005). Thus, our own face is of great value for our sense of identity. In modern society, people often modify their faces through the use of makeup, photo beautification, plastic surgery and other ways. One of the most common ways of retouching is to masculinize the face to make it look more handsome or to feminize the face to make it look more charming.

Masculinity/femininity refers to the traits or characteristics typically associated with being male/female, which is often associated with perceived facial attractiveness (Johnston, 2006; Perrett et al., 1998). Many studies have found that certain changes in sexual dimorphism can increase a face's attractiveness. For example, feminine female faces are often evaluated as being more attractive than masculine female faces (DeBruine et al., 2010; Fraccaro et al., 2010; Nakamura & Watanabe, 2019; Rhodes et al., 2000) since femininity in female faces is considered to be associated with health status and fertility (Baird et al., 1999; Law Smith et al., 2006; Thornhill & Gangestad, 1999). At the same time, the femininity of a face also induces the perception of some positive characteristics, such as warmth, honesty, cooperativeness, youthfulness, and parenting ability (Jones et al., 2011; Law Smith et al., 2006; Perrett et al., 1998; Welling et al., 2008). Some studies have found that feminine male faces are also regarded as more attractive (Jones et al., 2011; Nakamura & Watanabe, 2019; Perrett et al., 1998; Welling et al., 2008). This means that people seem to show a general preference for the femininity of faces, regardless of whether the faces are male or female (Carrito et al., 2018; Nakamura & Watanabe, 2019). However, other studies have found that people tend to prefer masculine male faces (DeBruine et al., 2006; Johnston et al., 2001; Little et al., 2008; Penton-Voak et al., 2001), as the individuals with masculine male faces are considered to have good genes and better disease resistance and immune response, which are beneficial for reproduction (Kirkpatrick & Ryan, 1991; Phalane et al., 2017; Rantala et al., 2013; Rhodes et al., 2003; Thornhill & Gangestad, 2006; Zahavi, 1975). A recent study (Li et al., 2020) has demonstrated that the influence of sexual dimorphism on perceived facial attractiveness also occurs in “self-face” evaluation. Specifically, when participants are presented with same-sex facial images, females evaluate their own original faces as being significantly more attractive than the masculinized and feminized self-faces, while males evaluate their own masculinized faces as being significantly more attractive than the feminized faces.

Although certain masculine/feminine changes to faces can increase facial attractiveness, they may affect the recognition of certain faces. For example, in one study, adults were unable to make accurate gender judgments of masculine female faces (Sugimura, 2006). In fact, individuals found it difficult to make gender judgements for both feminine male faces and masculine female faces (Deffenbacher et al., 1998). Conversely, faces modified toward their inherent features or archetype can be perfectly recognized (Yamaguchi et al., 1995). For example, Carrito et al. (2018) found that the gender identification of feminine female faces and masculine male faces was much better than that of masculine female faces and feminine male faces.

The abovementioned studies have indicated that masculine/feminine changes may increase the attractiveness of a face, but the extent of such change is not unconditional. To maintain the identity of faces, the modification of sexual dimorphism must be moderate. It has been demonstrated that facial identity is much more important than facial expressions in the judgment of attractiveness (Morrison et al., 2013). This is especially true for self-face evaluation. When people beautify their faces, they want other people to easily recognize that the face is their own, or the beautification is meaningless. However, no study to date has investigated the acceptable extent of masculine/feminine changes to individuals’ own faces in terms of attractiveness evaluation and the relationship of the attractiveness and similarity of a modified face with the original face. A recent study concerning voice attractiveness (Peng et al., 2020) found that the self-enhancement bias of voice attractiveness can only be generalized to similar and familiar versions of self-voice, thus has provided indirect evidence supporting the association of preference for self-information with identity.

In summary, the present study aims to explore the effect of masculine/feminine changes on the attractiveness evaluation of one's own face and the relationship of the attractiveness evaluation and similarity of the changed faces with the original self-face. To investigate whether the influence of sexual dimorphism on attractiveness evaluation is specific to self-face, we also adopt other faces as references, as was done in a previous study (Li et al., 2020). As the most familiar face and most salient representation of one's own identity (Gillihan & Farah, 2005; Keyes et al., 2010), we are more sensitive to our own faces. Consequently, people may be more critical of the masculine/feminine changes of their own faces than those of other faces.

## Method

### Participants

A total of sixty college students (30 females)^1^ from Zhejiang Sci-Tech University (Hangzhou, China) participated in this experiment in return for monetary compensation. The ages of females (19.37 ± 0.67 years old) and males (20.9 ± 2.35 years old) did not differ significantly (*p* > 0.05). All participants were right-handed with normal or corrected-to-normal vision. Written informed consent was obtained from each participant following a research protocol approved by the Institutional Review Board of the Department of Psychology at Zhejiang Sci-Tech University.

### Material

#### Self-face

Before the formal experiment, a facial image was obtained from each participant using an interchangeable-lens camera (SONY α 6000). To ensure that subjects’ expressions were neutral, they were first shown a standard neutral expression image. Participants were required to style their hair so that their entire face was exposed. In addition, they were asked to gaze directly at the camera and give a neutral expression. A photograph of their full face was taken at a distance of approximately 1.5 m.

**Figure 1.**
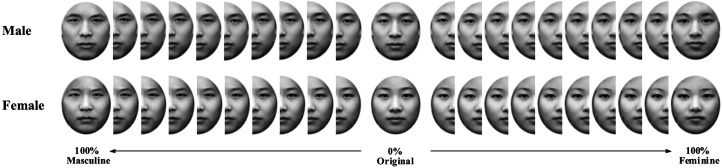
The facial sequence of the male other face and the female other face.

The original facial images were then standardized with Adobe Photoshop (https://www.adobe.com/products/photoshop.html). First, colored images were converted to black-and-white images. Second, the images were adjusted to have the same brightness. Third, the faces were cropped with an oval shape so that no hair or other information was included in the photos. The images were then resized to 350 × 414 pixels on a black background (see examples in Figure 1).

#### Other Faces

A male face and a female face were selected as other faces from the Cas-Peal face database (Gao et al., 2008). The selection process was as follows. First, 31 male and 31 female faces were randomly selected from the database. Then, thirty college students (15 females, 18–26 years old, mean: 23.17 ± 1.84 years old) who did not participate in the formal experiment rated the attractiveness of these faces on a 7-point Likert scale (1 = not at all attractive; 7 = very attractive). Based on the results, a male face and a female face with near-average attractiveness scores were finally selected as the other face stimuli. The two selected images were standardized with the same procedure as that used for the self-face.

#### Average Male and Female Faces

First, we selected 10 attractive male faces and 10 attractive female faces without makeup from resources on the Internet. Then, we created an average male and female face using Fantamorph 5 (https://www.fantamorph.com/) by synthesizing these 10 male or 10 female faces (cf., Little & Hancock, 2002).

#### Masculine and Feminine Transformation of Original Faces

Based on the original faces (i.e., self-faces, the male other face and female other face) and the average male and female face, we obtained the masculinized and feminized transformation of the original faces. First, 100% masculine and feminine faces were manufactured with the methods of Carrito et al. (2018) using Psychomorph 6 (http://users.aber.ac.uk/bpt/jpsychomorph/). That is, an original face was transformed 100% toward the direction of the average male face to obtain a “100% masculine face”, and an original face was transformed 100% toward the direction of the average female face to obtain a “100% feminine face”. Second, continuous morphed images were produced by parametrically blending the original images and the 100% masculine/feminine face using Fantamorph 5. For each type of original faces (self-faces, the male other face and female other face), we created 49 intermediate images, which changed in 2% incremental steps from the original face to the corresponding 100% masculine or feminine version. To this end, 101 images (1 original face, 50 masculinized faces and 50 feminized faces) were obtained for each type of original faces (see Figure 1 for the facial sequence of the male other face and the female other face).

### Procedure

Before the formal experiment, each participant took a photo of himself/herself, which was then standardized and transformed to make the formal experimental stimuli. One week later, the formal experiment began, which consisted of three tasks. Tasks 1 and 3 used E-Prime 2.0 (Psychology Software Tools, Inc., Pittsburgh, PA, USA) to show stimuli and record responses on the computer. Task 2 used Windows Media Player 12 (https://support.microsoft.com/) to present the stimuli, and participants answered questions on paper.

#### Task 1

In Task 1, twelve sequences of facial images were presented to each participant. Each sequence consisted of 51 continuously changed facial images. Each image was presented for 500 ms. This task consisted of two sessions. In one session, the facial sequence began with the 100% masculine or feminine face and then changed gradiently to the original face (0%). Participants were asked to press the space bar when they perceived the face presented on the screen as looking “like” the original face (himself/herself, the male other face or the female other face). In another session, the order of the facial sequence was reversed; i.e., it began with the original face (0%) and then changed gradiently to the 100% masculine or feminine face. Participants were required to press the space bar upon perceiving the face as looking “not like” the original face. The order of the two sessions, the sexual dimorphism (masculine, feminine) and the original face type (self-face, male other face, female other face) were counterbalanced across participants.

#### Task 2

In Task 2, the 6 facial sequences were presented in a video format with Windows Media Player 12. Half of the participants watched the videos changing from the original face to the 100% masculine or feminine face at a rate of 2%. The other half of participants watched the videos changing from the 100% masculine or feminine face to the original face. They were asked to choose the most attractive face in the videos and write it down on the answer sheet. When they chose, they could drag the progress bar forward or backward freely. The order of the original face type (self-face, the male other face, and the female other face) was counterbalanced across participants.

#### Task 3

Task 3 also consisted of two sessions. In session 1, participants were presented with six facial sequences. For half of the participants, the facial images in a sequence changed from the original face to the 100% masculine or feminine face at a rate of 10%; for the other half participants, the facial images changed from the 100% masculine or feminine face to the original face stepping by 10%. Each trial began with the fixation “ + ” for 500 ms, and then, a facial image appeared until a response was made. Participants were required to evaluate the attractiveness of the given face on a 7-point Likert scale (1: not at all attractive; 7: very attractive). In session 2, only the two self-face sequences were presented to the participants in a similar procedure as in session 1. Participants were required to evaluate the similarity of the given face with the original self-face on a 7-point Likert scale (1: not at all like myself; 7: very much like myself). The order of sessions 1 and 2 was counterbalanced across subjects.

Task 3 was conducted after Tasks 1 and 2. The order of Tasks 1 and 2 was counterbalanced across subjects.

### Data Analysis

In Task 1, the sequences of continuous facial images were presented either from the original face (self-face, the male other face, the female other face) to the corresponding 100% masculine/feminine face or in the opposite order. Consequently, we obtained two ratios representing the face “not like” the original face or that looks “like” the original face. Thus, the final ratio of a certain morphed face looking similar to an original face was defined as the average of these two data points. With the average ratios as the dependent variable, a 2 (evaluator gender: male, female) × 2 (sexual dimorphism: masculine, feminine) × 3 (face type: self, male_other, female_other) three-factor mixed-model ANOVA was conducted.

In Task 2, participants were asked to choose the most attractive face after watching a video clip of all the facial images in a sequence. The changing ratio, i.e., 0% (representing the original face) to 100% (representing the full masculine/feminine face) associated with the selected face, was regarded as the dependent variable, and a 2 (evaluator gender: male, female) × 2 (sexual dimorphism: masculine, feminine) × 3 (face type: self, male_other, female_other) three-factor mixed-model ANOVA was conducted.

In Task 3, participants rated the attractiveness of the faces ranging from the original faces to the corresponding 100% masculine or feminine face stepping by 10%. If a given face was regarded as being more attractive than the original face, then it meant that participants considered the face acceptable. Thus, we compared the attractiveness ratings of all the presented faces with those of the original faces (self-face, the male other face or female other face) with a paired t-test and obtained the acceptable changing range among which the associated attractiveness scores were significantly higher than or equal to that of the original face. Similarly, the acceptable changing range of the similarity of the morphed faces with the original face was calculated based on the similarity ratings in Task 3. Moreover, to investigate whether the attractiveness of a given face was based on this similarity judgment, correlation analyses between participants’ attractiveness scores and the similarity scores of the self-derived faces were conducted.

## Results

### Results of Task 1

In Task 1, two ratios were obtained. One was the transformation ratio when the corresponding face looked “not like” the original face when the facial sequence was presented from the original face to the 100% masculine/feminine face, indicating that the transformed faces beyond this ratio would no longer be regarded as being from the same person as that of the original face. The other ratio associated the face as beginning to look “like” the original face when the facial sequence was presented from the 100% masculine/feminine face to the original face, indicating that the transformed faces within this ratio would all be regarded as being from the same person as that of the original face. The mean of these two ratios was calculated to represent the average acceptable transformation extent of the original face to a 100% masculine/feminine face. These results are shown in Table 1.

**Table 1.** The Transformation Ratio of Sexual Dimorphism, Associating a Face as Being “Like” or “Not Like” the Original Face (*M* ± *SD*).

Evaluator gender	Sexual dimorphism	Self			m-Other			f-Other		
		like me	not like me	mean	like him	not like him	mean	like her	not like her	mean
Male	Masculine	0.33 ± 0.26	0.55 ± 0.21	0.44 ± 0.18	0.44 ± 0.30	0.71 ± 0.27	0.58 ± 0.20	0.38 ± 0.24	0.62 ± 0.23	0.50 ± 0.16
	Feminine	0.38 ± 0.26	0.59 ± 0.21	0.49 ± 0.19	0.34 ± 0.25	0.62 ± 0.25	0.48 ± 0.16	0.45 ± 0.29	0.71 ± 0.26	0.58 ± 0.20
Female	Masculine	0.28 ± 0.22	0.47 ± 0.16	0.37 ± 0.12	0.43 ± 0.27	0.69 ± 0.25	0.56 ± 0.20	0.40 ± 0.24	0.60 ± 0.22	0.50 ± 0.18
	Feminine	0.32 ± 0.22	0.58 ± 0.21	0.45 ± 0.15	0.34 ± 0.26	0.59 ± 0.22	0.46 ± 0.17	0.43 ± 0.29	0.66 ± 0.23	0.54 ± 0.18

*Notes*: m_Other: male other original face; f_Other: female other original face.

A 2 (evaluator gender: male, female) × 2 (sexual dimorphism: masculine, feminine) × 3 (face type: self, male_other, female_other) three-factor mixed-model ANOVA showed a significant main effect of face type (*F*(2, 116) = 14.513, *p* < 0.001, *η_p_^2^* = 0.200) and a significant interaction effect between sexual dimorphism and face type (*F*(2, 116) = 13.287, *p* < 0.001, *η_p_^2^* = 0.186), while no other significant main effect or interaction effect was found (all *p*s > 0.05).

**Figure 2.**
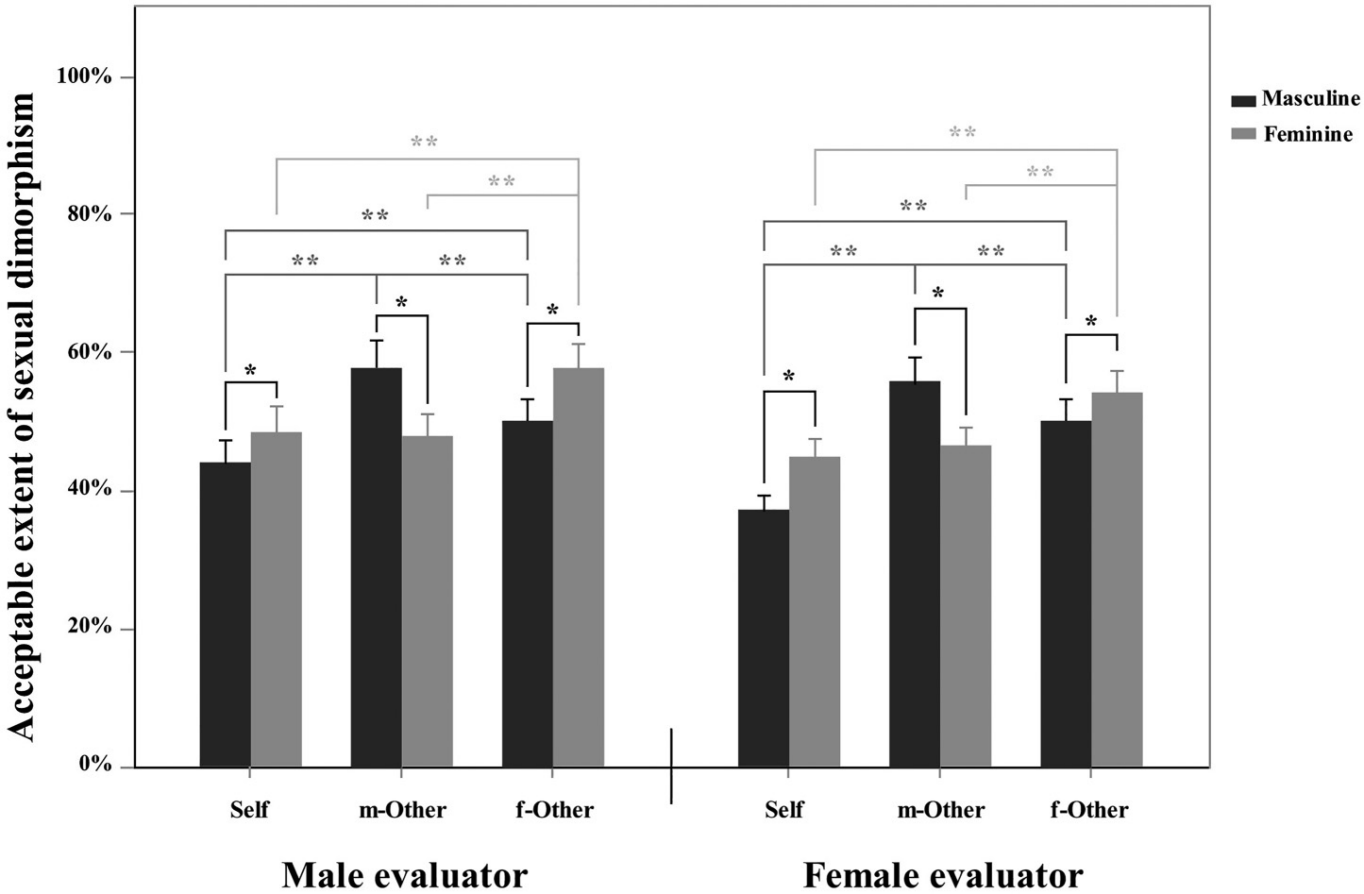
The acceptable transformation ratio of the original face (self-face, the male other face, and the female other face) to a 100% masculine/feminine face. Error bars indicate standard errors (SE).

The further analysis of simple effect revealed that for the self-face, the acceptable transformation ratio of the original face to the 100% feminine face was significantly higher than that to the 100% masculine face (*F*(1, 59) = 9.46, *p* = 0.003). For the male other face, participants were receptive to higher masculine changes than to feminine changes (*F*(1, 59) = 11.08, *p* = 0.002). In contrast, participants were receptive to higher feminine changes than to masculine changes for the female other face (*F*(1, 59) = 5.48, *p* = 0.023). On the other hand, for masculine changes, the order of the acceptable transformation ratio for the three types of faces was as follows: male_other > female_other > self (*F*(2, 118) = 19.94, *p* < 0.001), while for feminine changes, the acceptability was as follows: female_other > male_other and self (*F*(2, 118) = 8.22, *p* < 0.001). The Bonferroni correction was used in the simple effect analysis. See Figure 2 for an illustration.

m_Other: male other original face; f_Other: female other original face.

*p < 0.05; **p < 0.01.

### Results of Task 2

In Task 2, participants were required to select the most attractive face in the video clip of morphed faces. The corresponding transformation ratios of the selected faces are shown in Table 2.

**Table 2.** The Transformation Ratios of Sexual Dimorphism Corresponding to the Most Attractive Faces (*M* ± *SD*).

Evaluator gender	Sexual dimorphism	Self	m-Other	f-Other
Male	Masculine	0.21 ± 0.24	0.32 ± 0.28	0.22 ± 0.21
	Feminine	0.26 ± 0.23	0.44 ± 0.31	0.32 ± 0.26
Female	Masculine	0.25 ± 0.31	0.28 ± 0.19	0.23 ± 0.22
	Feminine	0.29 ± 0.25	0.49 ± 0.27	0.37 ± 0.28

*Notes*: m_Other: male other original face; f_Other: female other original face.

**Figure 3.**
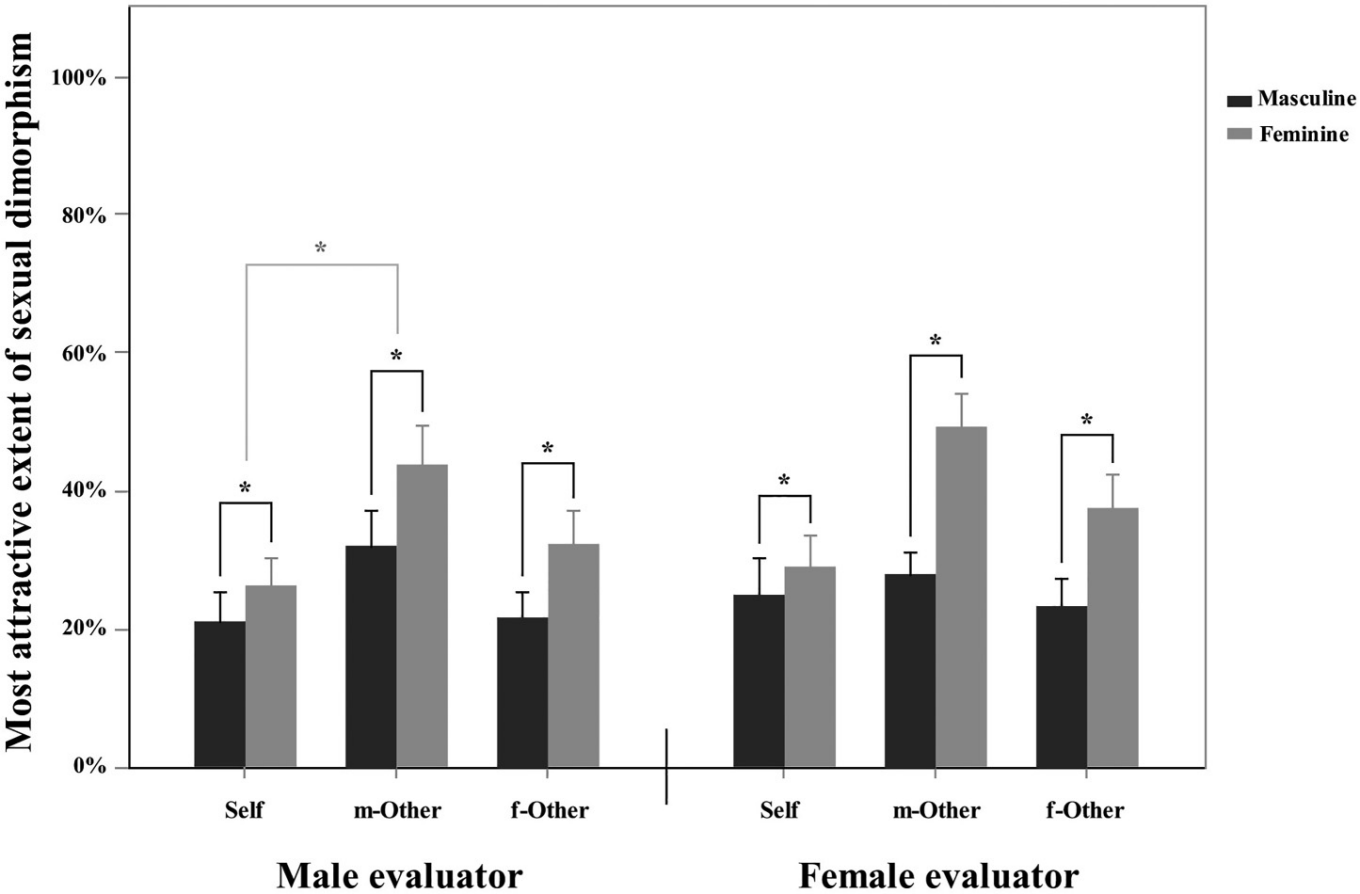
The transformation ratio of sexual dimorphism corresponding to the most attractive faces. Error bars indicate standard errors (SE).

A 2 (evaluator gender: male, female) × 2 (sexual dimorphism: masculine, feminine) × 3 (face type: self, male_other, female_other) three-factor mixed-model ANOVA yielded a significant main effect of sexual dimorphism (*F*(1, 58) = 14.018, *p* < 0.001, *η_p_^2^* = 0.195). The Bonferroni post hoc test revealed that participants regarded a higher ratio of feminized faces as being more attractive compared to masculinized faces. The main effect of face type was also significant (*F*(2, 116) = 9.998, *p* < 0.001, *η_p_^2^* = 0.147), indicating that participants evaluated a higher transformation ratio of male other face as being more attractive than self-face. No other main effect or interaction effect was found (all *p*s > 0.05). See Figure 3 for an illustration.

m_Other: male other original face; f_Other: female other original face.

*p < 0.05.

### Results of Task 3

The attractiveness rating scores of different masculine/feminine faces derived from the original faces (self-face, male other face or female other face) by male and female evaluators are shown in Table 3.

**Table 3.** The Attractiveness Rating Scores of Different Morphed Faces (*M* ± *SD*).

		Self		m-Other		f-Other	
		Male	Female	Male	Female	Male	Female
Masculine	100%	2.10 ± 1.32	2.70 ± 1.86	2.43 ± 1.25	1.80 ± 0.89	2.77 ± 1.28	3.13 ± 1.53
	90%	2.23 ± 1.30	2.80 ± 1.77	2.57 ± 1.17	1.77 ± 0.86	2.97 ± 1.25	2.97 ± 1.43
	80%	2.33 ± 1.21	2.87 ± 1.72	2.70 ± 1.26	2.10 ± 1.18	3.13 ± 1.25	2.97 ± 1.22
	70%	2.73 ± 1.41	3.13 ± 1.78	2.90 ± 1.35	2.27 ± 1.05	3.20 ± 1.19	2.93 ± 1.05
	60%	2.97 ± 1.47	3.27 ± 1.84	2.93 ± 1.26	2.53 ± 1.11	3.43 ± 1.25	3.00 ± 1.08
	50%	3.30 ± 1.74	3.20 ± 1.63	3.07 ± 1.34	2.63 ± 1.13	3.43 ± 1.36	3.53 ± 1.17
	40%	3.90 ± 1.86	3.43 ± 1.65	3.30 ± 1.32	2.67 ± 1.18	3.67 ± 1.56	3.77 ± 1.17
	30%	4.20 ± 1.75	3.60 ± 1.48	3.43 ± 1.22	2.97 ± 1.27	3.90 ± 1.09	4.10 ± 1.21
	20%	4.50 ± 1.46	4.13 ± 1.50	3.50 ± 1.41	3.00 ± 1.51	4.03 ± 1.03	4.23 ± 1.25
	10%	4.67 ± 1.52	4.47 ± 1.55	3.43 ± 1.38	3.20 ± 1.49	4.40 ± 1.28	4.57 ± 1.17
Original	0%	4.70 ± 1.51	4.50 ± 1.38	3.40 ± 1.45	3.17 ± 1.42	4.27 ± 1.44	4.77 ± 1.25
Feminine	10%	4.47 ± 1.39	4.43 ± 1.52	3.53 ± 1.38	3.00 ± 1.31	4.47 ± 1.50	4.80 ± 1.03
	20%	4.27 ± 1.39	4.67 ± 1.42	3.50 ± 1.20	2.80 ± 1.21	4.40 ± 1.35	4.63 ± 1.19
	30%	4.23 ± 1.41	4.47 ± 1.33	3.20 ± 1.37	2.87 ± 1.36	4.20 ± 1.40	4.50 ± 1.33
	40%	3.73 ± 1.41	4.23 ± 1.41	3.20 ± 1.27	2.53 ± 1.20	4.13 ± 1.33	4.47 ± 1.57
	50%	3.40 ± 1.30	4.00 ± 1.68	2.83 ± 1.21	2.47 ± 1.28	4.00 ± 1.51	4.23 ± 1.55
	60%	2.87 ± 0.94	3.47 ± 1.38	2.77 ± 1.25	2.33 ± 1.15	3.70 ± 1.53	4.13 ± 1.48
	70%	2.60 ± 1.16	3.20 ± 1.35	2.63 ± 1.38	2.33 ± 1.27	3.67 ± 1.65	3.97 ± 1.50
	80%	2.37 ± 1.22	2.73 ± 1.23	2.37 ± 1.38	2.37 ± 1.56	3.60 ± 1.69	3.70 ± 1.62
	90%	2.40 ± 1.35	2.43 ± 1.30	2.10 ± 1.16	2.10 ± 1.35	3.40 ± 1.63	3.47 ± 1.63
	100%	2.27 ± 1.36	2.30 ± 1.32	1.87 ± 1.04	2.00 ± 1.14	2.93 ± 1.46	3.10 ± 1.65

*Notes*: m_Other: male other original face; f_Other: female other original face.

**Figure 4.**
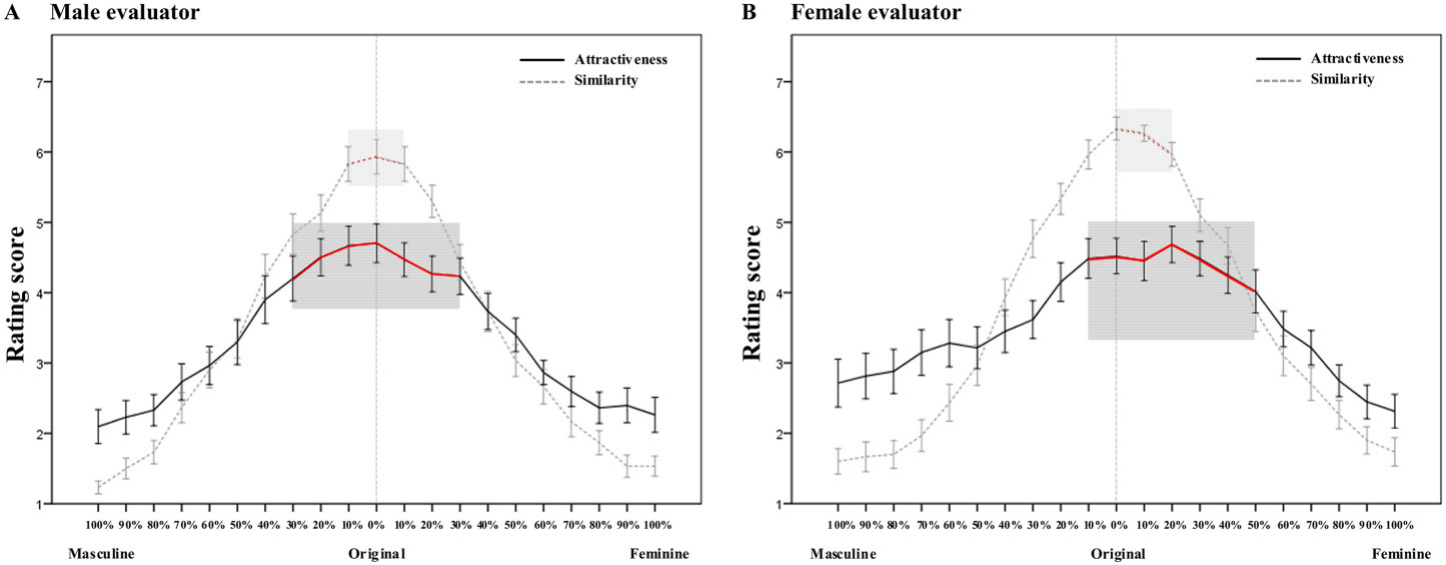
The rating scores of the attractiveness (solid line) and similarity (dashed line) of different morphed faces from the original self-face to 100% masculine/feminine face by male evaluators (A) and female evaluators (B). The red line in the shaded area indicates the acceptable range of attractiveness/similarity within which the rating score is significantly higher than or equal to that of the original self-face. Error bars indicate standard errors (SE).

**Figure 5.**
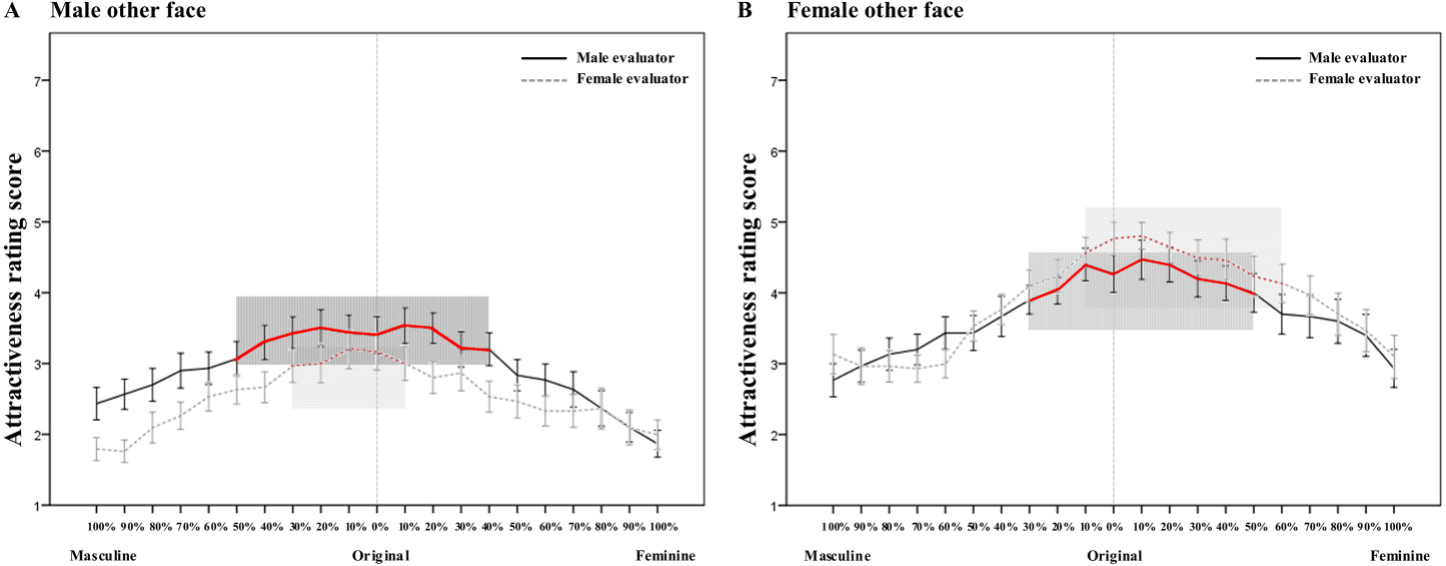
The rating scores of the attractiveness of the different morphed faces from the original male other face (A) or female other face (B) to a 100% masculine/feminine face by male evaluators (solid line) and female evaluators (dashed line). The red line in the shaded area indicates the acceptable range of attractiveness within which the rating score is significantly higher than or equal to that of the original face. Error bars indicate standard errors (SE).

When the attractiveness rating of a given morphed face was significantly higher than or equal to that of the original face, it was regarded as acceptable in terms of attractiveness. Thus, we obtained the acceptable changing range for the three types of facial sequences. For the sequence derived from the original self-face, the acceptable range was [*Masculine* 30% - *Feminine* 30%] by male evaluators (see the red solid line in Figure 4A for details) and [*Masculine* 10% - *Feminine* 50%] by female evaluators (see the red solid line in Figure 4B for details). For male other face sequences, the acceptable range was [*Masculine* 50% - *Feminine* 40%] by male evaluators (see the red solid line in Figure 5A for details) and [*Masculine* 30% - *Feminine* 10%] by female evaluators (see the red dashed line in Figure 5A for details). For female other face sequences, the acceptable range was [*Masculine* 30% - *Feminine* 50%] by male evaluators (see the red solid line in Figure 5B for details) and [*Masculine* 10% - *Feminine* 60%] by female evaluators (see the red dashed line in Figure 5B for details).

The similarity rating scores of different masculine/feminine faces with the original self-face by male and female evaluators are shown in Table 4.

**Table 4.** The Similarity Rating Scores of Different Morphed Self-faces With the Original Self-face.

Evaluator gender		Masculine										Original	Feminine									
		100%	90%	80%	70%	60%	50%	40%	30%	20%	10%	0%	10%	20%	30%	40%	50%	60%	70%	80%	90%	100%
Male	*Mean*	1.23	1.50	1.73	2.37	2.90	3.33	4.23	4.83	5.13	5.83	5.93	5.83	5.30	4.43	3.73	3.03	2.67	2.17	1.87	1.53	1.53
	*SD*	0.50	0.82	0.91	1.19	1.40	1.45	1.70	1.56	1.41	1.34	1.34	1.34	1.26	1.38	1.55	1.25	1.35	1.18	0.94	0.86	0.78
Female	*Mean*	1.60	1.67	1.70	1.97	2.43	2.97	3.93	4.77	5.33	5.97	6.33	6.27	5.97	5.10	4.67	3.73	3.10	2.70	2.27	1.90	1.73
	*SD*	1.15	1.15	1.09	1.25	1.43	1.56	1.44	1.45	1.21	1.13	0.88	0.64	0.93	1.27	1.42	1.57	1.54	1.29	1.11	1.06	1.11

When the similarity rating of a given morphed face was significantly higher than or equal to that of the original self-face, it was regarded as being acceptable in terms of similarity. The acceptable changing range for the facial sequence was [*Masculine* 10% - *Feminine* 10%] by male evaluators (see the red dashed line in Figure 4A for details) and [*Masculine* 0% - *Feminine* 20%] by female evaluators (see the red dashed line in Figure 4B for details).

**Figure 6.**
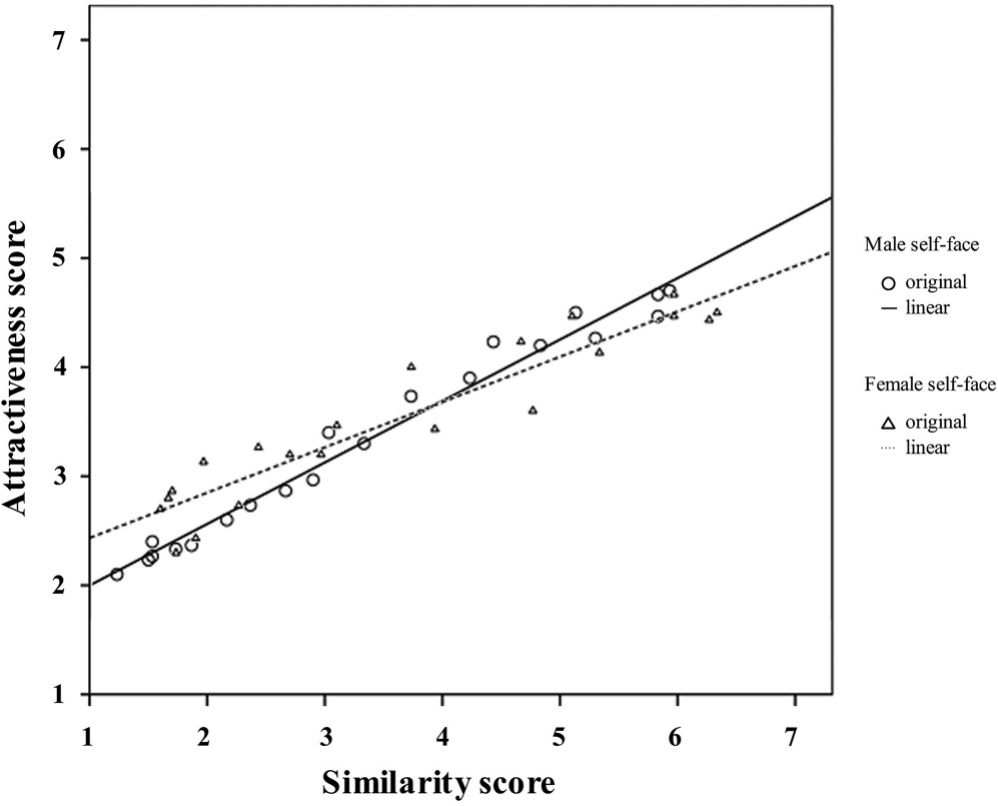
The correlation between the rating scores of the similarity and attractiveness of the morphed male self-face (solid line) and female self-face (dashed line).

A Pearson correlation analysis of the attractiveness and similarity scores for the morphed self-face revealed that the correlations were significant for both male self-face (*r* = 0.989, *p* < 0.001) and female self-face (*r* = 0.943, *p* < 0.001) (see Figure 6 for an illustration). This means that faces with higher similarity to the original self-face were evaluated as being more attractive.

## Discussion

The present study manipulated the sexual dimorphism of self-faces and other faces, i.e., transforming the original faces to different masculinized or feminized extents, and explored the influence of the modification of sexual dimorphism on the attractiveness evaluation and similarity judgment. The results showed that participants considered a higher ratio of femininized faces to be more attractive than that of masculinized faces. For the male other face, masculine changes tended to be accepted, while feminine changes were accepted for the female other face. More importantly, the acceptable range of masculine/feminine transformation for self-faces was smaller than that for other faces. In addition, the attractiveness ratings for morphed self-faces were correlated with the similarity scores between the faces and the original self-faces.

The results of Task 2 revealed that when selecting the most attractive faces, participants chose a higher ratio of femininized faces than masculinized faces for both self-faces and other faces, irrespective of whether evaluators were male or female (see also Figure 3). This finding is consistent with previous studies; that is, people exhibited a general preference for the femininity of faces, regardless of whether the faces were male or female (Carrito et al., 2018; Kočnar et al., 2019; Nakamura & Watanabe, 2019; Nakamura & Watanabe, 2020; Perrett et al., 1994; Saxton et al., 2011). This may be due to people's positive perceptions of femininity. Individuals with feminine faces tend to have more ideal personality traits than do those with masculine faces (Oh et al., 2020; Scott et al., 2008). Feminine facial features (such as large, round eyes, a smaller chin and fuller lips) indicate warmth, honesty, cooperation and parental qualities (Berry & McArthur, 1985). However, the masculinization of faces increases feelings of aggression and dominance (Swaddle & Reierson, 2002), and individuals with such faces are regarded as being more violent and callous and less honest and cooperative (Boothroyd et al., 2007; Johnston et al., 2001). This result is further consolidated by the finding that participants can accept a greater extent of feminized changes than masculinized changes; i.e., faces with a higher ratio of feminization is regarded as still being “like” the original faces (i.e., self-face and female other face) compared to those of masculinization, regardless of whether the evaluator was male or female (see also Figure 2).

The results of Task 1 showed that regarding the acceptable extent of sexual dimorphism (i.e., the ratio of a certain morphed face still looking similar to the original face), the ratio of masculine face is higher than that of the feminine face for the male other facial sequence, while the ratio of feminine face is higher than that of masculine face for the female other face. This gender consistency effect occurs regardless of evaluator gender (see also Figure 2). This means that for other faces, people are more likely to accept the change in the faces in a dimorphic direction toward the original gender. In other words, people are more likely to accept changes in male faces toward the masculine direction and accept changes in female faces toward the feminine direction. This is in line with the results of previous studies (Carrito et al., 2018; Li et al., 2020). For example, Carrito et al. (2018) found that participants responded faster when evaluating the attractiveness of masculinized male faces compared to feminized male faces, and participants were more accurate when discriminating the gender of feminized female faces than masculinized female faces. More direct supporting evidence from Li et al. (2020) showed that male participants evaluated their own masculine faces as being significantly more attractive than feminine faces in the same-sex context. Such a gender consistency effect could also be reflected in the acceptable range of attractiveness. Specifically, for male other faces, the acceptable range is biased to the greater extent of masculinity, while for female other faces, the acceptable range is biased to the greater extent of femininity, regardless of the gender of evaluators (see also Figure 5). This gender consistency effect might be explained by the prototype effect in facial recognition (Bartleft et al., 1984; Cabeza et al., 1999).

The results of Task 3 showed that when evaluating the attractiveness of masculine/feminine other faces, male participants (contrary to females) exhibited a broader acceptable range within which the attractiveness score was significantly higher than or equal to that of the original faces. That is, male participants regarded 50% masculine transformation and 40% feminine transformation to the male other face as acceptable, while the acceptable range of female participants was between 30% masculine transformation and 10% feminine transformation. For the female other face, the acceptable range for male participants was [Masculine 30% - Feminine 50%], contrary to [Masculine 10% - Feminine 60%] for female participants (it is worth noting that female participants accepted greater (60%) feminine transformation for the female other face due to the gender consistency effect; see also Figure 5). This pattern was also present in the results of self-faces; i.e., men have a considerable acceptable range of both masculine and feminine transformation (i.e., [Masculine 30% - Feminine 30%]), while women are receptive to more feminized transformation ([Masculine 10% - Feminine 50%]) (see also Figure 4 for details). These results were consistent with the findings of previous studies that people have a certain degree of acceptance of masculine male faces (DeBruine et al., 2006; Johnston et al., 2001; Little et al., 2008; Penton-Voak et al., 2001) and feminine male faces (Jones et al., 2011; Nakamura & Watanabe, 2019; Perrett et al., 1998; Welling et al., 2008). However, females tend to accept the feminine transformation of their own faces (DeBruine et al., 2010; Fraccaro et al., 2010; Nakamura & Watanabe, 2019; Rhodes et al., 2000). Regarding the acceptable range of similarity for the self-face, male participants showed acceptance of both masculine and feminine transformation (i.e., [Masculine 10% - Feminine 10%]), while female participants accepted only feminine transformation (i.e., [Masculine 0% - Feminine 20%]). Although the pattern was similar to that of the attractiveness rating, the acceptable range was much narrower, indicating that people were more sensitive to and harsher about the modification in their identity (i.e., similarity in the present study) than in attractiveness evaluation. The broader acceptable range of masculine/feminine transformation by males compared to females may be explained by the fact that men usually focus on status and ability, while women tend to pay attention to physical appearance (Luxen & Van De Vijver, 2006).

Regarding the acceptable range of attractiveness, it is narrower for self-faces than for other faces (see also Figures 4 and 5). These results are further corroborated by the results of Task 1; that is, the acceptable transformation ratio of the original self-face is significantly lower than that of other faces. This finding indicates that people are more sensitive to their own faces and thus more fastidious to masculine/feminine changes of the self faces. As a self-face is the most familiar face and most salient representation of one's own identity (Gillihan & Farah, 2005; Keyes et al., 2010), people are very sensitive to changes in their own faces (Brooks & Kemp, 2007; Buttle & Raymond, 2003; O’Donnell & Bruce, 2001). For example, people recognize their own faces faster and more accurately than strangers’ faces (Keyes et al., 2010), and they can recognize their own faces with fewer attentional resources (Alzueta et al., 2019). As a result, changes in the self-face would lead to greater changes in identity (Kelly, 1992). In addition, people are more sensitive to displacements of the internal components (e.g., eyes and nose) of familiar faces compared to unfamiliar faces (Brooks & Kemp, 2007). This is consistent with the fact that people are more efficient at scanning familiar faces (Heisz & Shore, 2008) and could also explain the findings in Task 3, that the acceptable range of similarity is much narrower than that of attractiveness (see also Figure 4).

The results of the correlation analysis in our study revealed that participants’ attractiveness ratings of the morphed self-faces were significantly correlated with the ratings of the similarity between such faces and the original self-face. This suggests that the more similar a face is to the original self-face, the more attractively the face will be perceived. This result is in line with previous studies (Bruno et al., 2013; Sulutvedt & Laeng, 2014). For example, Bruno et al. (2013) found that when choosing the most attractive facial image of their romantic partner among several variants, participants preferred a “self-based morph” (i.e., their partner's face blended with 22% of their self-face) to other morphed images. The effect of self-resemblance appeared even when compared with the morph of their partner's face blended with their partner's same-sex “prototype” (which was judged as being more attractive than the self-face by other individuals). Sulutvedt and Laeng (2014) found similar results: female participants preferred self-based morphs to prototype faces. An explanation for the higher attractiveness associated with more similar faces is the “mere exposure effect” (Moreland & Zajonc, 1982), which is a psychological phenomenon in which people tend to favor familiar things (Bornstein & D’Agostino, 1992). In the present study, the original self-face was the most familiar face for participants (Alzueta et al., 2019; Keyes et al., 2010), and the closeness of a modified face to the original self-face could represent the familiarity of that face; thus, faces that are more similar to the original self-face lead to higher perceptions of attractiveness among participants.

In our daily lives, people, especially females, often want to refine their faces to achieve higher attractiveness via cosmetics, retouching, plastic surgery, or facial beautification (e.g., using picture editing software) to make themselves look more masculine or feminine. However, our findings suggest that masculine/feminine modifications of faces must be conducted with great caution. When beautifying your face through masculinizing or feminizing yourself, you must still “be yourself”.

In conclusion, the present results show that the acceptable range of masculine/feminine transformation for self-faces is smaller than that for other faces. Furthermore, the attractiveness ratings for masculinized or femininized self-faces are correlated with the similarity scores between the faces and the original self-faces. These findings contribute to a better understanding of how humans perceive sexually dimorphic modifications in terms of attractiveness judgments and provide important implications for beautification through masculinity/femininity.

## Notes

1. An a priori sample size estimate was calculated by using G*Power 3.1 (Faul et al., 2009; Prajapati et al., 2010). When using an alpha level of 0.05 and statistical power of 0.95 with an effect size f of 0.20, we found a required sample size of more than 56 total participants.
